# National French Survey of Coronavirus Disease (COVID-19) Symptoms in People Aged 70 and Over

**DOI:** 10.1093/cid/ciaa792

**Published:** 2020-06-18

**Authors:** Cédric Annweiler, Guillaume Sacco, Nathalie Salles, Jean-Pierre Aquino, Jennifer Gautier, Gilles Berrut, Olivier Guérin, Gaetan Gavazzi

**Affiliations:** 1 Department of Geriatric Medicine and Memory Clinic, Research Center on Autonomy and Longevity, University Hospital, Angers, France; 2 UPRES EA 4638, Université d’Angers, Angers, France; 3 Robarts Research Institute, Department of Medical Biophysics, Schulich School of Medicine and Dentistry, the University of Western Ontario, London, Ontario, Canada; 4 Department of Clinical Gerontology, University Hospital, Bordeaux, France; 5 Délégation générale de la Société Française de Gériatrie et Gérontologie (SFGG), Paris, France; 6 Pôle hospitalo-universitaire de gérontologie clinique, CHU de Nantes, France; 7 Université Côte d’Azur, Centre Hospitalier Universitaire de Nice, Service de Médecine Gériatrique et Thérapeutique, Nice, France; 8 Université Côte d’Azur, CNRS UMR 7284/INSERM U108, Institute for Research on Cancer and Aging Nice (IRCAN), Faculté de médecine, Nice, France; 9 Service Gériatrie Clinique, Centre Hospitalo-Universitaire Grenoble-Alpes, Saint-Martin-d’Hères, France

**Keywords:** COVID-19, SARS-Cov-2, semiology, symptomatology, older adults

## Abstract

The objective of this national French survey was to determine the coronavirus disease 2019 (COVID-19) semiology in seniors (n = 353; mean, 84.7 ± 7.0 years). A total of 57.8% of patients exhibited ≤3 symptoms, including thermal dysregulation (83.6%), cough (58.9%), asthenia (52.7%), polypnea (39.9%), and gastrointestinal signs (24.4%). Patients ≥80 years exhibited falls (*P* = .002) and asthenia (*P* = .002). Patients with neurocognitive disorders exhibited delirium (*P* < .001) and altered consciousness (*P* = .001). Clinical peculiarities of COVID-19 were reported in seniors.

**Clinical Trials Registration:**

NCT04343781.

Since December 2019, coronavirus disease 2019 (COVID-19) caused by severe acute respiratory syndrome coronavirus-2 (SARS-CoV-2) is spreading worldwide from China, affecting millions of people. Although older adults do not appear more prone than younger ones to be infected, they are more at risk of developing severe and lethal forms of COVID-19 [1–3]. The core question is thus to properly discuss the diagnosis of COVID-19 in older patients. It is commonly admitted that the semiology of older adults differs from that encountered in younger ones. Changes in the clinical expression of the diseases and difficulties in interpreting the clinical signs in older patients could blur the diagnosis process. If these peculiarities were also retrieved with COVID-19, it could be the cause of delayed diagnosis among older patients and be responsible for delayed care and isolation measures with subsequent higher risk of virus propagation. The objective of this national French survey was to describe and identify the symptoms most frequently encountered in people aged 70 years and older diagnosed with COVID-19.

## METHODS

This cross-sectional study was conducted by the French Society of Geriatrics and Gerontology. An online standardized questionnaire was sent by email to all Society members and widely communicated through the professional networks in geriatrics and infectious diseases. Physicians were asked to report, between March 22 and April 5, 2020, their last 10 patients aged ≥70 years with confirmed SARS-CoV-2 infection (as defined as a positive reverse transcriptase-polymerase chain reaction [RT-PCR] test result). Those who had treated fewer than 10 diagnosed patients were asked to submit a questionnaire for each of them. The study was conducted in accordance with the ethical standards set forth in the Helsinki Declaration (1983), was declared to the National Commission for Information Technology and civil Liberties (ar20-0031v1), and was registered on clinicaltrials.gov under number NCT04343781.

The following characteristics were collected for each patient: demographic (age, sex, place where living, place of care, most recent disability score according to the Iso-Resource Group)[4], and medical history (major neurocognitive disorders [MND], hypertension, diabetes mellitus, asthma or chronic obstructive pulmonary disease, cardiomyopathy, severe chronic renal failure defined as creatinine clearance <30 mL/min, solid or hematological cancer).

The following symptoms observed within the first 72 hours of SARS-CoV-2 infection (ie, 72 hours from suspicion, possibly before diagnostic confirmation by RT-PCR test) were collected for each patient using yes/no questions: general signs (sudden deterioration of general condition, temperature, blood pressure), respiratory signs (cough, polypnea), ear nose and throat signs (rhinorrhea, odynophagia, otalgia, conjunctivitis, dysgeusia or ageusia, anosmia), gastrointestinal signs (diarrhea, nausea, vomiting) and geriatric syndromes (falls, hypo- or overactive delirium, altered consciousness). Changes in complete blood count (leukopenia, lymphopenia, thrombocytopenia) were also collected, with details when available.

Qualitative variables were described using numbers and percentages, and quantitative variables using means and standard deviations. Comparisons between participants aged ≥80 years and <80 years, and between those with and without MND, were performed using χ ^2^ test for qualitative variables (or exact Fisher test where appropriate), and Student *t* test for quantitative variables (or Mann-Whitney *U* test where appropriate). Univariate logistic regressions were conducted to determine the association of each COVID-19 sign with age ≥80 years and history of MND. Finally, the profiles of COVID-19 patients were determined according to their symptoms, age, and history of MND using a multiple correspondence analysis. Two-sided *P* values < .05 were considered significant. Analyses were performed with SAS (SAS Institute Inc.; v9.4) and R (R Core Team 2020; v3.6.3) using the FactoMineR and Factoshiny packages.

## RESULTS

Older patients’ characteristics are presented in Table 1 (N = 353; mean ± SD, 84.7 ± 7.0 years; 54.7% women). Most patients (57.8%) exhibited ≤3 symptoms, and 15% had 0–1 symptom during the first 72 hours of the infection. The most frequent symptoms were thermal dysregulation (83.6%), cough (58.9%), and sudden deterioration of general condition (52.7%). Polypnea was found in 39.9% including n = 47 with severe polypnea ≥ 30 cycles/min, and gastrointestinal signs in 24.4% including n = 77 with diarrhea. Biologically, 76.4% of the population had lymphopenia, with 725 ± 267 lymphocytes per mm^3^ on average.

**Table 1.** Characteristics and Comparisons of Participants (N = 353) Separated According to Their Age and History of MNDs

	Population of the Study (N = 353)	Comparison of Patients < and > 80 y of Age			Comparison of Patients With and Without MNDs		
		Age < 80 y (n = 89)	Age ≥ 80 y (n = 264)	*P* Value^a^	No MND (n = 219)	With MND (n = 134)	*P* Value^a^
**Sociodemographic data**							
Male	160 (45.3)	47 (52.8)	113 (42.8)	.101	105 (48.0)	55 (41.0)	.206
Age (y), mean ± SD	84.7 ± 7.0	75.4 ± 2.9	87.8 ± 4.8		83.7 ± 7.2	86.3 ± 6.4	**<.001**
Place where living				**.0049**			
Community-dwelling	257 (72.8)	75 (84.3)	182 (68.9)		180 (82.2)	77 (57.5)	**<.001**
Institution-dwelling	96 (27.2)	14 (15.7)	82 (31.1)		39 (17.8)	57 (42.5)	
Place of care				.1845			.173
Hospital	324 (91.8)	79 (88.8)	245 (92.8)		204 (93.2)	120 (89.6)	
Nursing home	23 (6.5)	8 (9.0)	15 (5.7)		11 (5.0)	12 (9.0)	
Services residence	3 (0.9)	0 (0.0)	3 (1.1)		1 (0.5)	2 (1.5)	
Personal residence	3 (0.9)	2 (2.3)	1 (0.4)		3 (1.4)	0 (0.0)	
GIR^b^				**<.001**			**<.001**
1	23 (7.1)	4 (5.0)	19 (7.7)		1 (0.5)	22 (17.3)	
2	70 (21.5)	11 (13.8)	59 (24.0)		13 (6.5)	57 (44.9)	
3	47 (14.4)	8 (10.0)	39 (15.9)		24 (12.1)	23 (18.1)	
4	59 (18.1)	8 (10.0)	51 (20.7)		40 (20.1)	19 (15.0)	
≥ 5	127 (39.0)	49 (61.3)	78 (31.7)		121 (60.8)	6 (4.7)	
**Medical history**							
Major neurocognitive disorders	134 (38.0)	21 (23.6)	113 (42.8)	**.001**	0 (0.0)	134 (100.0)	
Hypertension	234 (66.3)	50 (56.2)	184 (69.7)	**.012**	144 (65.8)	90 (67.2)	.786
Diabetes mellitus	80 (22.7)	23 (25.8)	57 (21.6)	.407	52 (23.7)	28 (20.9)	.535
Asthma or COPD	46 (13.0)	13 (14.6)	33 (12.5)	.610	32 (14.6)	14 (10.5)	.259
Cardiomyopathy	159 (45.0)	24 (27.0)	135 (51.1)	**<.001**	92 (42.0)	67 (50.0)	.143
Severe chronic renal failure	38 (10.8)	5 (5.6)	33 (12.5)	.070	22 (10.1)	16 (11.9)	.577
Solid or hematological cancer	67 (19.0)	15 (16.9)	52 (19.7)	.554	45 (20.6)	22 (16.4)	.337
**General signs**							
Sudden deterioration of general condition	186 (52.7)	34 (38.2)	152 (57.6)	**.002**	113 (51.6)	73 (54.5)	.599
Low blood pressure	51 (14.5)	8 (9.0)	43 (16.3)	.090	31 (14.2)	20 (14.9)	.842
Body temperature				**.011**			**.013**
No fever	58 (16.4)	14 (15.7)	44 (16.7)		36 (16.4)	22 (16.4)	
Subfebrile temperature 37.5°–38°C	75 (21.3)	10 (11.2)	65 (24.6)		35 (16.0)	40 (29.9)	
Hyperthermia > 38°C	198 (56.1)	62 (69.7)	136 (51.5)		135 (61.6)	63 (47.0)	
Alternation of hyperthermia and hypothermia	22 (6.2)	3 (3.4)	19 (7.2)		13 (5.9)	9 (6.7)	
**Respiratory signs**							
Cough	208 (58.9)			.153			**<.001**
Sputum	82 (23.2)	14 (15.7)	68 (25.8)		49 (22.4)	33 (24.6)	
Dry	126 (35.7)	35 (39.3)	91 (34.5)		97 (44.3)	29 (21.6)	
Polypnea	141 (39.9)			.165			**.014**
Between 23 and 29/min	94 (26.6)	17 (19.1)	77 (29.2)		58 (26.5)	36 (26.9)	
≥ 30/min	47 (13.3)	12 (13.5)	35 (13.3)		38 (17.4)	9 (6.7)	
**ENT signs**							
Rhinorrhea	32 (9.1)	9 (10.1)	23 (8.7)	.691	23 (10.5)	9 (6.7)	.229
Odynophagia	9 (2.6)	2 (2.3)	7 (2.7)	1.000	7 (3.2)	2 (1.5)	.492
Otalgia	2(0.6)	1 (1.1)	1 (0.4)	.441	2 (0.9)	0 (0)	.528
Conjunctivitis	3 (0.9)	1 (1.1)	2 (0.8)	1.000	1 (0.5)	2 (1.5)	.560
Dysgeusia—ageusia^c^	25 (7.1)	8 (9.0)	17 (6.5)	.428	22 (10.1)	3 (2.3)	**.006**
Anosmia^c^	7 (2.0)	0 (0)	7 (2.7)	.198	6 (2.8)	1 (0.8)	.260
**Gastrointestinal signs**							
Diarrhea	77 (21.8)	23 (25.8)	54 (20.5)	.287	53 (24.2)	24 (17.9)	.165
Nausea—vomiting	22 (6.2)	3 (3.4)	19 (7.2)	.197	16 (7.3)	6 (4.5)	.286
**Geriatric syndromes**							
Falls	66 (18.7)	7 (7.9)	59 (22.4)	**.002**	36 (16.4)	30 (22.4)	.164
Hypoactive delirium	62 (17.6)	14 (15.7)	48 (18.2)	.599	25 (11.4)	37 (27.6)	**<.001**
Overactive delirium	32 (9.1)	5 (5.6)	27 (10.2)	.190	12 (5.5)	20 (14.9)	**.003**
Altered consciousness	37 (10.5)	6 (6.7)	31 (11.7)	.183	14 (6.4)	23 (17.2)	**.001**
**Biology**							
Lymphopenia^d^	247 (74.6)	66 (78.6)	181 (73.3)	.336	165 (79.3)	82 (66.7)	**.011**
Lymphocytes/mm^3^ (n = 246), mean ± SD	725 ± 267	730 ± 285	723 ± 261	.856	692 ± 258	791 ± 275	**.016**
Leukopenia^e^	9 (2.7)	2 (2.4)	7 (2.8)	1.000	5 (2.4)	4 (3.2)	.733
Leukocytes/mm^3^ (n = 17), mean ± SD	2 394 ± 881	2613 ± 1174	2327 ± 818	.571	2261 ± 1091	2545 ± 603	.630
Thrombopenia^f^	85 (25.4)	24 (28.6)	61 (24.3)	.436	58 (27.6)	27 (21.6)	.244
Thrombocytes/mm^3^ (n = 84), mean ± SD	115 523 ± 29 085	113 708 ± 32 512	116 250 ± 27 860	.984	112 810 ± 31 743	121 577 ± 21 354	.355

Data presented as n (%) where applicable. *P* < .05 indicated in bold.

^a^Comparisons based on χ ^2^ test or exact Fisher test for qualitative variables, and Student *t* test or Mann-Whitney *U* test for quantitative variables, as appropriate.

^b^27 missing data.

^c^2 missing data.

^d^22 missing data.

^e^20 missing data.

^f^18 missing data.

Abbreviations: COPD, chronic obstructive pulmonary disease; ENT, ear nose and throat; GIR, Iso-Resource Group; MND, major neurocognitive disorders; SD, standard deviation.

Comparison of those < 80 and > 80 years of age showed that falls (22.4% vs 7.9%, *P* = .002; odds ratio [OR] = 3.37; 95% confidence interval [95% CI], 1.48–7.69) and sudden deterioration of general condition (57.6% vs 38.2%, *P* = .002; OR = 2.20; 95% CI, 1.34–3.59) were more frequently after 80 years of age, whereas fever was less frequent (51.5% vs 69.7%, *P* = .011; OR = 0.46; 95% CI, .28–.77).

Comparison between patients with and without MND showed that those with MND exhibited more often hypoactive (27.6% vs 11.4%, *P* < .001; OR = 2.96; 95% CI, 1.69–5.20) and overactive delirium (14.9% vs 5.5%, *P* = .003; OR = 3.03; 95% CI, 1.43–6.42) and altered consciousness (17.2% vs 6.4%, *P* = .001; OR = 3.03; 95% CI, 1.50–6.13), and less often hyperthermia (47.0% vs 61.6 %, *P* = .013; OR = 0.55; 95% CI, .36–.85), cough (46.3% vs 66.7%, *P* < .001; OR = 0.35; 95% CI, .21–.57), and dysgeusia-ageusia (2.3% vs 10.1%, *P* = .006; OR = 0.21; 95% CI, .06–.70).

Finally, the multiple correspondence analysis results distinguished between 2 profiles of older patients. The first profile matched with patients younger than age 80 years without MND, who exhibited more frequent hyperthermia and cough during the first 72 hours of the infection, but no fall, altered consciousness, or hypoactive delirium. In contrast, the second profile matched with patients aged 80 years and older with MND; the latter exhibiting more frequently no specific symptoms, and most often an absence of hyperthermia, polypnea, cough, and dysgeusia-ageusia.

## Discussion

This national French survey shows that older adults with COVID-19 exhibit a pauci-symptomatic clinical picture with less than 3 signs during the first 72 hours of the infection, generally combining general and respiratory signs (eg, hyperthermia and cough) with peculiarities that should alert the clinician (eg, sudden deterioration of general condition, diarrhea, lymphopenia, geriatric syndromes including falls and delirium). Various clinical profiles were highlighted across older adults, especially among the oldest-old ≥80 years and those with chronic diseases such as MND.

Our survey provides the first description of the COVID-19 signs in older, and even oldest-old, adults with comorbidities [1–3]. Compared with previous meta-analyses in younger adults [5–7], we found that older adults with COVID-19 often exhibit thermal dysregulation, which, however, results less often in hyperthermia (56% here vs 82% [5] to 91% [6] in younger adults) and more often in subfebrile temperatures or alternations of hyperthermia and hypothermia (not described thus far to our knowledge). The prevalence of cough was similar (59% here vs 61% [5] to 72% [7] in younger adults). In contrast, the sudden deterioration of general condition, mostly illustrated by marked asthenia, was particularly frequent in older adults (53% here vs 36% [5] to 51% [6] in younger adults). Also, older adults exhibited more often dyspnea (40% here vs 26% [5] to 30% [6] in younger adults) and gastrointestinal signs (24.4% here with mostly diarrhea [21.8%] vs 10% in younger adults [5, 8]). This should encourage clinicians to integrate the gastrointestinal signs into the diagnostic reasoning for SARS-CoV-2 infection in older adults. Older adults had less often anosmia (2% here vs 86% in younger adults [9]) and dysgeusia-ageusia (7% here vs 89% in younger adults [9]). The latter prevalence should be cautiously interpreted however because of olfactory and gustatory dysfunctions with advancing age [10]. Finally, we found a higher proportion of lymphopenia in older adults compared with the general population (75% here vs 55% [3]). Lymphopenia was more significant than that usually observed in the normal aging population (750/mm^3^ vs 1432/mm^3^ in the literature [11]), and may explain part of the excess mortality observed in older adults with COVID-19 [1].

Our study has a number of limitations. This is an observational cross-sectional study conducted on a panel of French older patients who may be not representative of the general older population. The 64 physicians who responded to the survey, however, came from all French regions. A reporting bias cannot be ruled out because the accuracy and completeness of the data were entirely reliant upon physicians’ declarations, although the questionnaire was designed to limit variability in readers’ interpretations by asking only factual data. Also, in the absence of mass screening policy in France, only patients for whom a biological test had been carried out because of suspected infection, for clinical reasons for example, could be included, which may have overestimated the prevalence of some signs. The lack of control group prevented to determine the average number of symptoms met in non-COVID-19 French older adults. Similarly, no data were available on the use of concomitant drugs, for example of antibiotics, which could partially explain increases in gastrointestinal signs. Finally, only patients diagnosed with RT-PCR test were included, although the sensitivity of this test presents a relatively high risk of false negatives (sensitivity of 72%) [12], which may have excluded a number of patients with COVID-19.

In conclusion, this national French survey revealed that the clinical picture of older adults with COVID-19 includes both general and respiratory signs like in younger adults (eg, hyperthermia and cough), but also more peculiar features such as marked asthenia, diarrhea, lymphopenia, and geriatric syndromes. We also reported various clinical profiles across older adults, notably in those aged 80 years and older and those with a history of MND who appeared particularly pauci- or asymptomatic during the first 72 hours of the infection. These findings should be integrated into the clinical reasoning in geriatric medicine and encourage the systematization of diagnostic tests for SARS-CoV-2 infection in older adults.

## Notes

***Author contributions.*** C. A. has full access to all of the data in the study, takes responsibility for the data, the analyses, and interpretation, and has the right to publish any and all data, separate and apart from the attitudes of the sponsors. All authors have read and approved the manuscript. Study concept and design: C. A., G. S., N. S., G. B., O. G., and G. G. Acquisition of data: C. A., G. S., N. S., J. P. A., J. G., G. B., O. G., and G. G. Analysis and interpretation of data: C. A., G. S., and J. G. Drafting of the manuscript: C. A., G. S., and J. G. Critical revision of the manuscript for important intellectual content: N. S., J. P. A., G. B., O. G., and G. G. Statistical expertise: J. G. Administrative, technical, or material support: C. A. Study supervision: C. A.

***Acknowledgments.*** The authors thank all participants and services for their cooperation; Melinda Beaudenon, MS, and Romain Simon, MS, from the Research Center on Autonomy and Longevity, University Hospital of Angers, France; Caroline Pastorelli, from the French Society of Geriatrics and Gerontology (SFGG); and the COVID-19 study group of the SFGG. SFGG COVID-19 study group: Didier Albert, Cédric Annweiler, Gaëlle Annweiler, Jean-Pierre-Aquino, Marine Asfar, Adeline Bannier, Jean Barré, Axel Bernard, Gilles Berrut, Mohand Chérif Bessai, Yves Beucher, Maëlle Beunardeau, Isabelle Biajoux, Clémence Boully, Anne-Sophie Boureau, Antoine Brangier, Thomas Brunet, Hélène Campana Briault, Edouard Chaussade, Adrien Cohen, Julian Cornaglia, Axelle Courau, Nicolas Crochette, Sylvie Dardalhon, Guillaume Duval, Marine de La Chapelle, Séverine Deledicq, Mathilde Devaux, Gael Durel, Élise Fiaux, Thibaut Fraisse, Xavier Galimard, Jennifer Gautier, Gaetan Gavazzi, Béatrice Gonzalez, Olivier Guérin, Aline Gury, Valérie Haguenauer-Bariteau, Julie Hamrit, Céline Hervo, Béatrice Lacave, Maxime Le Floch, Gwenaël Le Moal, Pierre-Marie Liais, Matthieu Lilamand, Jocelyne Loison, Paul Loubet, Karin Maley, Hélène Mangeard, Géraldine Martin-Gaujard, Marc Mennecart, Hélène Meytadier, Jean-Marc Michel, Nathalie Michel-Laaengh, Elena Paillaud, Mathieu Priner, Emeline Proye, Guillaume Sacco, Nathalie Salle, Denis Soriano, Maturin Tabue Teguo, Julien Vernaudon, François Weill, and Julien Zirnhelt. Permission has been obtained from all persons named in this section. No participant objected to the use of anonymized clinical and biological data for research purposes.

***Disclaimer*.** Patient level data are freely available from the corresponding author at Cedric.Annweiler@chu-angers.fr. There is no personal identification risk within this anonymized raw data, which is available after notification and authorization of the competent authorities.

***Potential conflicts of interest.*** The authors: No reported conflicts of interest. All authors have submitted the ICMJE Form for Disclosure of Potential Conflicts of Interest.
